# Supplementary material for: AMPK-Mediated Phosphorylation of Nrf2 at S374/S408/S433 Favors Its βTrCP2-Mediated Degradation in KEAP1-Deficient Cells
Source: Antioxidants (Basel). 2023 Aug 9;12(8):1586. doi: 10.3390/antiox12081586 (PMC10451539; doi:10.3390/antiox12081586)
Supplement: Supplementary file 1 [file antioxidants-12-01586-s001.zip › antioxidants-2531422-supplementary.pdf]

A

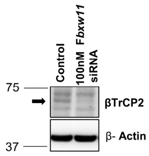

Figure S1. Validation of the *Fbxw11* knock down in *Keap1-/-* cells. A. *Keap1-/-* MEFs were transfected with 100 nM control or *Fbxw11*-specific siRNA for 48 h. Cell lysates were subjected to immunoblot analysis for endogenous βTrCP2 or β-Actin. A representative blot (A) is depicted. The arrow indicates the band of endogenous βTrCP2.
